# Dynamics of prevalence and diversity of avian malaria infections in wild *Culex pipiens* mosquitoes: the effects of *Wolbachia*, filarial nematodes and insecticide resistance

**DOI:** 10.1186/1756-3305-7-437

**Published:** 2014-09-16

**Authors:** Flore Zélé, Juilen Vézilier, Gregory L’Ambert, Antoine Nicot, Sylvain Gandon, Ana Rivero, Olivier Duron

**Affiliations:** Maladies Infectieuses et Vecteurs: Ecologie, Génétique, Evolution et Contrôle, (UMR CNRS-UM1-UM2 5290, IRD 224), Centre de Recherche IRD, 911 Avenue Agropolis, 34394 Montpellier, France; Institut des Sciences de l’Evolution, (UMR CNRS 5554), Université de Montpellier 2, 34095 Montpellier, France; Centre d’Ecologie Fonctionnelle et Evolutive, (UMR CNRS 5175), 1919 Route de Mende, 34293 Montpellier, France; Entente départementale pour la Démoustication du littoral méditerranéen, 165 avenue Paul-Rimbaud, 34184 Montpellier, France; Centro de Biologia Ambiental, Faculdade de Ciencias da Universidade de Lisboa, Edificio C2, 30 Piso Campo Grande, 1749016 Lisbon, Portugal

**Keywords:** *Culex pipiens*, Avian malaria, *Plasmodium*, *Wolbachia*, Filarial nematodes, Insecticide resistance

## Abstract

**Background:**

Identifying the parasites transmitted by a particular vector and the factors that render this vector susceptible to the parasite are key steps to understanding disease transmission. Although avian malaria has become a model system for the investigation of the ecological and evolutionary dynamics of *Plasmodium* parasites, little is still known about the field prevalence, diversity and distribution of avian *Plasmodium* species within the vectors, or about the extrinsic factors affecting *Plasmodium* population dynamics in the wild.

**Methods:**

We examined changes in avian malaria prevalence and *Plasmodium* lineage composition in female *Culex pipiens* caught throughout one field season in 2006, across four sampling sites in southern France. Using site occupancy models, we correct the naive estimates of *Plasmodium* prevalence to account for PCR-based imperfect detection. To establish the importance of different factors that may bear on the prevalence and diversity of avian *Plasmodium* in field mosquitoes, we focus on *Wolbachia* and filarial parasite co-infections, as well as on the insecticide resistance status of the mosquito.

**Results:**

*Plasmodium* prevalence in *Cx. pipiens* increased from February (0%) to October (15.8%) and did not vary significantly among the four sampling sites. The application of site occupancy models leads to a 4% increase in this initial (naive) estimate of prevalence. The parasite community was composed of 15 different haemosporidian lineages, 13 of which belonged to the *Plasmodium* genus, and 2 to the *Haemoproteus* genus. Neither the presence of different *Wolbachia* types and of filarial parasites co-infecting the mosquitoes, nor their insecticide resistance status were found to affect the *Plasmodium* prevalence and diversity.

**Conclusion:**

We found that haemosporidian parasites are common and diverse in wild-caught *Cx. pipiens* mosquitoes in Southern France. The prevalence of the infection in mosquitoes is unaffected by *Wolbachia* and filarial co-infections as well as the insecticide resistant status of the vector. These factors may thus have a negligible impact on the transmission of avian malaria. In contrast, the steady increase in prevalence from February to October indicates that the dynamics of avian malaria is driven by seasonality and supports that infected birds are the reservoir of a diverse community of lineages in southern France.

**Electronic supplementary material:**

The online version of this article (doi:10.1186/1756-3305-7-437) contains supplementary material, which is available to authorized users.

## Background

In the last few decades, avian malaria has become a model system for the investigation of the ecological and evolutionary dynamics of *Plasmodium* parasites in the wild [1–4]. These studies have allowed the identification of more than 900 lineages (as defined by their cytochrome-b sequence) in over 600 bird species distributed all over the world [5]. Some of these lineages are extremely prevalent in particular geographical areas (upwards of 90% [3, 6]) and able to infect a wide range of hosts, while others are rarer and confined to a particular species or family [5, 7]. Comparatively little is known about the prevalence and distribution of avian *Plasmodium* species within the vectors in the field [4, 6–13]. Only a fraction of the known avian *Plasmodium* lineages have been matched to a putative vector and, thus far, only ca. 20 mosquito vector species have been identified (MalAvi Database). Vectors, however, play a key role in dynamics and epidemiology of the disease. Vector populations fluctuate temporally and spatially, and these processes generate variability in the host biting rate, which ultimately bear on the parasite prevalence and population dynamics of the infection [14]. In addition, vectors play a key role in structuring host-parasite relationships by, for example, restricting the access of certain parasites to a particular subset of hosts (but see [2, 8]) or by limiting or blocking the transmission of parasites to which the vector is less susceptible [15, 16]. Identifying the parasites transmitted by a particular vector and the factors that render this vector susceptible to the parasite are therefore key steps to understanding the epidemiology of the disease.

The aim of the present study is two-fold. The first aim is to establish whether there are variations across space and time in the prevalence and diversity of avian malaria infections in *Culex pipiens* mosquitoes, the main vector of avian malaria in Europe [6, 7, 10]. For this purpose, we sampled *Cx. pipiens* mosquitoes for ten consecutive months on four different locations within the Rhone delta (France) and we looked for differences in prevalence and diversity of avian malaria infections across space and time. One pervasive, but rarely acknowledged, problem of parasite prevalence studies in vectors is imperfect detection. Even with modern and *a priori* more sensitive PCR-based detection techniques, the probability of detection of parasites is strongly correlated with parasite load. Low parasite loads can be easily missed, leading to an underestimation of the parasite’s prevalence [17, 18]. This problem is particularly acute for vectors that feed on birds because of interference between the parasite’s DNA and the DNA from the nucleated erythrocytes. To address this issue, our prevalence estimates were corrected by using site occupancy models. These models are based on the repeated sampling of each “site” (in our case, a site corresponds to a single mosquito) in order to obtain an estimate of the probability of detection of the parasite (*p*), which is then used to correct the observed prevalence [17, 19]. Site-occupancy models are commonly used in ecology to estimate the density and range of species distributions (e.g. [20–22]) but are still seldom used in the field of host-parasite interactions [17, 18] and have, to our knowledge, never been applied to malaria vectors.

The second aim is to establish the importance of different factors that may bear on the prevalence of avian *Plasmodium* in *Cx. pipiens* mosquitoes in the field. In the wild, vectors are rarely infected by a single parasite. More often than not, a suite of microorganisms ranging from virus and bacteria to protozoan, and even metazoan, parasites can be found competing with each other for space and nutritional resources within the vector [23–26]. As a result, recent years have seen a growing interest on the role of such co-infections in shaping the epidemiology of vector-transmitted diseases. Here, we focus specifically on the presence of *Wolbachia* and filarial parasite co-infections on avian *Plasmodium* prevalence and diversity in the field. *Wolbachia pipientis,* a maternally inherited intracellular bacterium, is the most common microorganism in insects. In recent years there has been a plethora of studies showing that *Wolbachia* interferes with the development of a wide range of pathogens [27, 28]. Studies conducted on *Plasmodium*, however, suggest that the outcome of the co-infection is largely dependent on the particular *Wolbachia-Plasmodium* combination used: some combinations seem to inhibit [29–33] while others facilitate [32, 34, 35] the parasite’s development. To our knowledge, however, no study has investigated the role that *Wolbachia* infections may play in structuring *Plasmodium* infections in field-caught mosquitoes. *Wolbachia* infections are near to or at fixation in *Cx. pipiens* populations worldwide [36, 37], where it is responsible for complex patterns of cytoplasmic incompatibility, a type of conditional sterility between hosts harboring incompatible infections [38]. In the Montpellier region, *Cx. pipiens* populations harbor a considerable diversity of *Wolbachia* strains belonging to three different phylogenetic groups: *w* Pip-I, *w* Pip-II and *w* Pip-III [37, 39, 40]. Therefore, although no *Wolbachia-* uninfected *Cx. pipiens* mosquitoes exist in nature that would allow testing the effect of *Wolbachia* presence/absence on the probability of being infected with *Plasmodium*, this system provides an interesting opportunity to investigate the role of *Wolbachia* diversity on *Plasmodium* prevalence in the field. Filarial infections have also been shown to influence the prevalence and intensity of infection of *Plasmodium* in mosquitoes [23, 41]. In Southern Europe, *Cx. pipiens* is the main vector of *Dirofilaria immitis,* a filarial parasite of humans and cannids [42–44], but there is a paucity of data on the prevalence and co-occurrence of *Plasmodium* and filarial infections in this mosquito species.

The region where the study took place has been repeatedly treated with organophosphate insecticides for the last 40 years. As a result, the prevalence of insecticide resistance in *Cx. pipiens* mosquitoes in the Montpellier region is high [45, 46]. It has been suggested that the evolution of insecticide resistance in mosquitoes entails a series of drastic physiological and immunological changes that may potentially alter their ability to transmit diseases [47]. Using data obtained both in the laboratory and in the field, McCarroll *et al.*[48, 49] have indeed shown that insecticide resistant *Cx. quinquefasciatus* mosquitoes are less likely to transmit the filarial parasite *Wuchereria bancrofti.* The limited evidence available from *Plasmodium* is, however, contradictory and comes exclusively from the laboratory: while one study found no effect of insecticide resistance on *Plasmodium* prevalence or intensity [50], a later study found that insecticide resistance increases the susceptibility of mosquitoes to *Plasmodium*[51]. Our sampling provided an unparalleled opportunity to investigate whether the insecticide resistance status of mosquitoes bears on the prevalence or diversity of *Plasmodium* in wild-caught mosquitoes. For this purpose, we typed mosquitoes for the two main types of insecticide resistance present in the area: target site resistance (through the modification of the acetylcholinesterase [52]) and metabolic resistance (through the overproduction of detoxifying carboxylesterases [53]).

By following avian malaria infections in *Cx. pipiens* mosquitoes for ten consecutive months and across four different sites, our study aimed to address several relevant but hitherto seldom explored determinants of avian malaria prevalence and diversity in wild populations of mosquitoes, namely: 1) Does *Plasmodium* prevalence and diversity vary across space and time in the area of study? 2) Can site occupancy models be used to detect and correct a bias in the estimation of *Plasmodium* prevalence in mosquitoes? 3) Do mosquitoes bearing a particular strain of *Wolbachia* have a higher probability of being infected by avian malaria? 4) What is the prevalence of filarial infections in the area and are these infections correlated with the prevalence of *Plasmodium*? and 5) Are insecticide resistant mosquitoes more or less likely to transmit avian malaria than their susceptible counterparts and, if so, is this correlation associated with a particular insecticide resistant mechanism (detoxification vs target site modification)?

## Methods

### Study areas and mosquito sampling

**Figure 1:**
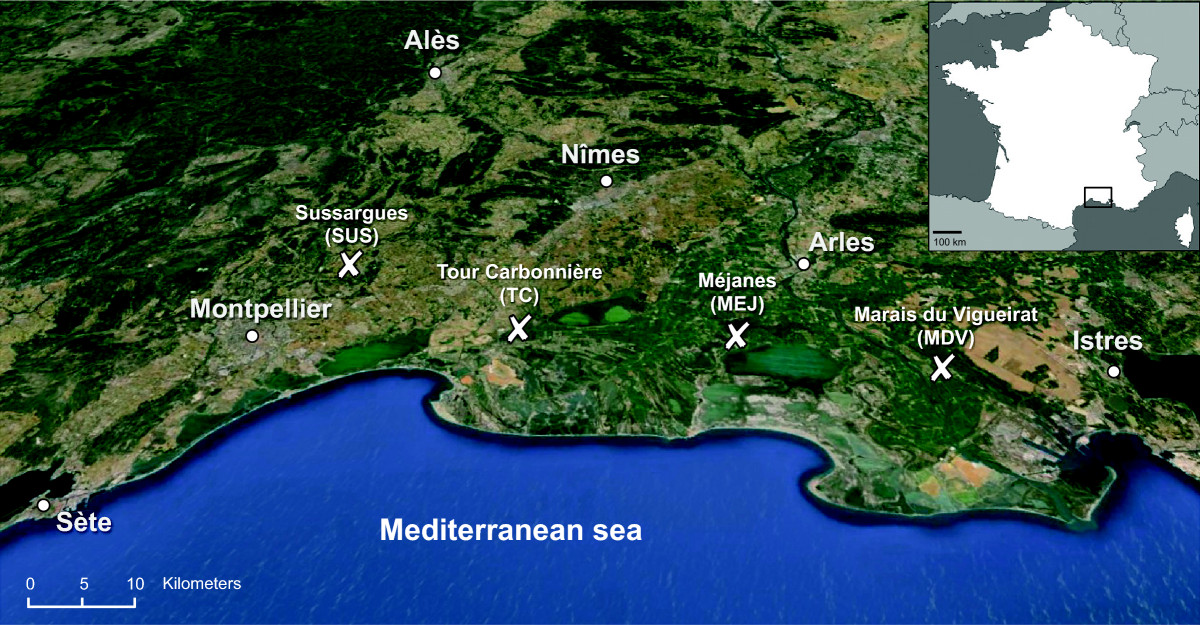
**Map showing sampling sites (crosses) where** ***Culex pipiens*** **mosquitoes were collected in the Rhône delta in South of France.** Map: GOOGLE EARTH - Data SIO, NOAA, U.S. Navy, NGA, GEBCO, © 2013 Google Landsat Image.

The study was carried out in four sample sites in the Rhône delta, along a North-West to South-East transect (43°42’07”- 43°30’20”N, 4°00’33”- 4°47’29” E, Figure 1) which mostly consisted of wetlands (ponds, marshes, paddies, reed beds and swamps). Sample sites are roughly 23 km away from each other: the Sussargues site (SUS: 43°42’07” N, 4°00’33”E, elevation 50 m) contains Mediterranean forest, scrubland, and stone quarries, with few habitations; Tour Carbonnière (TC: 43°36’28” N, 4°13’49”E, elev. 0 m) is located close to the village of Saint Laurent d’Aigouze where rice fields, ponds and reed beds dominate; in Méjanes (MEJ: 43°34’13”N, 4°30’02”E, elev. 3 m), rice fields, reed beds and marshes with meadows and horses are abundant; finally, Marais du Vigueirat is a natural bird reserve (MDV: 43°30’20”N, 4°47’29” E, elev. 0 m) and consists of marshes, swamps, paddies and reed beds. *Culex pipiens* females were trapped every fortnight, for two consecutive nights (from 18:00 to 10:00), over a 10 month period (February 20th to December 1st) in 2006. Two pigeon-baited traps hung on trees (protected from sunlight and wind exposure) were used per site, one in the canopy (5–10 meters height, depending on the vegetation) and the other one close to the ground. A detailed description of the pigeon-baited traps used here is given in L’Ambert *et al*. [54]. This method allows the sampling of host-seeking mosquitoes and maximizes the chances of collecting non-blood-fed females. As a precaution, however, the absence of remnant bird blood in the digestive tract was controlled for by observing under a binocular microscope (mosquitoes with blood meal were removed from further analyses). Eliminating blood fed mosquitoes reduced the chances of false positives because of parasites in the blood meal inside the gut. A total of 1156 unfed *Cx. pipiens* mosquitoes (identified using morphological characteristics [55]) were collected in this way.

### Detection of avian malaria parasites

**Figure 2:**
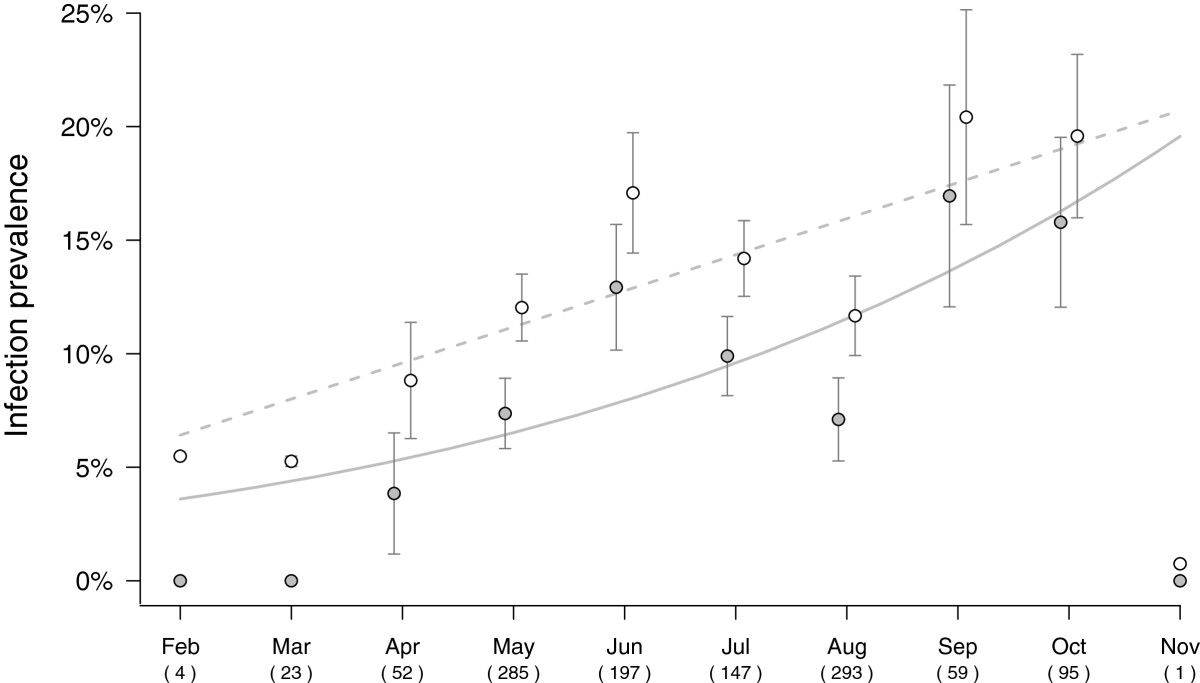
**Prevalence of haemosporidian infection over time.** Naïve mosquito prevalence over the ten month sampling period is plotted as gray-filled circles, corrected prevalence (calculated using site-occupancy models) as empty circles. Gray and dashed lines indicate the predicted values for naïve and estimated infection prevalence respectively using a GLM models with a logit link (binomial errors). Numbers between brackets indicate the total number of mosquitoes sampled at each time point.

Total DNA was extracted from each individual mosquito (n = 1156) using the QIAGEN protocol and materials (DNeasy 96 Tissue Kit, Qiagen NV, Venlo, The Netherlands) and total DNA was eluted in the final step with 80 μL RNase free water (Qiagen). The DNA quality was systematically tested using a PCR amplification of a fragment of the *Cx. pipiens cytb* as described in [56]. Avian malaria parasites were detected for each mosquito sampled by using the nested PCR method developed by [57], which amplifies a 448 bp fragment of the haemosporidia *cytb* gene. Infected-positive individuals were used as positive controls in each PCR assay. This technique allows the detection of haemosporidian parasites belonging to the genus *Plasmodium* but also *Haemoproteus*. This method detected haemosporidian parasites in 98 individual mosquitoes (henceforth “haemosporidian pool”) across a set of geographic locations and collection dates (see Figures 1 and 2). In order to obtain a non-infected “control pool”, we randomly sampled (using RANDOM.ORG; http://www.random.org/) 140 haemosporidian*-* negative individuals uniformly distributed across the same geographic locations and collection sites.

### Estimation of avian malaria parasite prevalence

Haemosporidian detection rates have been shown by qPCR to be strongly dependent on host parasite load: low parasite loads can be easily mistaken for lack of infection leading to an underestimation of prevalence [17, 18]. To correct for false negatives and thereby obtain a more accurate estimate of prevalence, we applied a site-occupancy modeling framework to the dataset [19, 58]. Site occupancy models are based on the repeated sampling of each individual “site” (in our case a “site” corresponds to an individual mosquito) in order to obtain an estimate of the probability of detection or, in other words, an estimate of the sensitivity of the test (i.e. false negatives, see [17] for more conceptual and methodological information). For this purpose, we repeated the nested PCRs an average of 6 times for the individuals from the “haemosporidian pool” (n = 98), and an average of 8 times for the individuals from the “control pool” (n = 140). In the “haemosporidian pool”, 66.3% of the samples always gave a positive result across the 6 PCRs, 13.3% showed variable patterns of positive/negative results, and 20.4% were positive only once. After this intense sampling effort, 12 individuals originally assigned to the “control pool” (n = 128 after reassignment) were re-assigned to the “haemosporidian pool” (n = 110 after reassignment). False positive diagnoses were assumed not to occur (the nested PCRs systematically used a negative control). This method gave us what we call ‘naïve prevalence’ hereafter.

We fitted models using the software PRESENCE version 3.1 [59] and we used the Akaike Information Criterium (AIC; see [60]), to select the best fit model (“1 group constant probability”). The number of sampling occasions was set to 12 (the maximum number of PCRs carried out on a single individual) since the program controls for missing data. The individual detection probabilities given by the model were used to correct the prevalence of haemosporidians per location and sampling month: called ‘estimated prevalence’ hereafter.

### Phylogenetic analysis of avian *Plasmodium* parasites

Molecular identification and phylogenetic analysis of avian malaria parasites was carried out using 96 samples randomly picked from the *“* haemosporidian pool” (96 is the number of wells in a standard PCR plate). Fragments (at least 448-bp-long) of the *cytb* gene were sequenced in forward sense, using the internally nested primer HAEMF from products of the initial PCR [57]. Sequences were edited and aligned using the program ClustalW included in the software MEGA version 5.1 beta [61] with additional manual editing. Mitochondrial DNA lineages were blasted against known avian malaria lineage sequences available in the MalAvi database [5] and in GenBank. The chromatograms were also checked for double nucleotide peaks to infer possible cases of mixed infections of two different parasite lineages. The sequences were assigned to an already described lineage only if they were identical to a reference sequence present in the MalAvi database. For the lineages differing by one nucleotide from the known lineage the term “-like” was appended to the name (e.g. “SGS1-like”) for the phylogenetic analyses but, for simplicity, they were considered to be the same lineage for all other analyses. New sequences have been deposited in GenBank (see Table 1) and whenever the information was available, lineages were then assigned to a given morphospecies using the MalAvi database (see Table 1).

**Table 1:** **Information relative to the haemosporidians lineages found in this study**

Lineages	MorphoSpieces	Mosquito vectors	Bird hosts order	Region	GenBank	References
SGS1	*P. relictum*	*Cx. pipiens*	Passeriformes, Galliformes, Gruiformes, Procellariiformes, Sphenisciformes	Europe, Asia,	AF495571	[1, 4, 6, 11, 62–65]
		*Cx. pipiens pallens*		Africa, Australia, Pacific,		
		*Cx. modestus*		S-America		
		*Cx. sasai*				
		*Cx. theileri*				
		*Lutziavorax*				
DELURB4	*Plasmodium spp.*	*-*	Passeriformes	Europe	EU154346	[66]
DELURB5	*Plasmodium spp.*	*Cx. perexiguus*	Passeriformes	Europe	EU154347	[11, 66]
		*Cx. theileri*				
PADOM01	*-*	*Cx. pipiens*	Passeriformes	Europe, Asia, C-America	DQ058611	[4, 67]
GRW06	*P. elongatum*	*-*	Passeriformes, Strigiformes, Coraciiformes	Europe, Asia, Africa, Australia, N- and C-America	DQ368381	[68, 69]
			Ciconiiformes, Columbiformes			
SYAT05	*P. vaughani*	*Cx. pipiens*	Passeriformes	Europe, Australia, N-America, Pacific	DQ847271	[1, 4, 6, 11, 70]
		*Cx. restuans*	Columbiformes			
		*Cx. pipiens pallens*				
COLL1	*P. relictum*	*Cx. pipiens*	Passeriformes	Europe, Asia	AY831747	[4, 71]
CXPIPS1	-	*-*	-	-	KJ579150	this study
CXPIPS2	*-*	*-*	-	-	KJ579151	this study
GRW11	*P. relictum*	*Cx. quinquefasciatus*	Passeriformes, Galliformes	Europe, Asia, Africa	AY831748	[4, 6, 62, 71, 72]
		*Cx. pipiens pallens*				
		*Cx. pipiens*				
LINN1	*Plasmodium spp.*	*Cx. restuans*	Passeriformes	Europe, Australia, N-America	DQ847270	[73–76]
		*Cx. pipiens*				
PADOM01 like	*-*	*-*	-	-	KJ579153	this study
SGS1 like	*-*	*-*	-	-	KJ579152	this study
CXPIPS3	*Haemoproteus spp.*	*-*	-	-	KJ579154	this study
GAGLA03	*Haemoproteus spp.*	*-*	Passeriformes	Europe	GU085197	[77]

Lineages are classified according to their prevalence. Morphospecies or parasite genus, mosquito vectors, bird hosts order and sample regions are given according to the MalAvi database. For each lineage, we give the GenBank accession number used to build the phylogeny. Independently, we also give a non-exhaustive list of references in which the different lineages can be found, with emphasis on studies of vectors.

We compared 448-bp-long fragments of the sequences obtained from this study with five published sequences that have been reliably identified to *Plasmodium* morphospecies level and/or that are as close as possible to the new sequences obtained in this study: CXQUI01, GRW04, MANSON01, PADOM05 and SYAT03 [GenBank: AB308051, AF254975, AB308052, EU708328 and AY831752, respectively]. We rooted our tree with four mammalian malaria parasites sequences [78]. The program jModelTest version 0.1.1 [79] indicated that the most likely model of sequence evolution was TIM2 + G. We used Maximum-Likelihood analysis implemented in PhyML (v.3.0) [80] to reconstruct a phylogeny using these parameters and the software FigTree v1.3.1 was used to draw the phylogenetic tree. Node supports in the resulting phylogeny were tested using 5000 bootstrap replications.

### Molecular identification of *Wolbachia* and filarial nematode

*Wolbachia* and filarial genotyping were performed on 100 randomly chosen mosquitoes from the “haemosporidian pool” and 92 randomly chosen mosquitoes from the “control pool”. *Wolbachia* genotyping was performed by analyzing the polymorphism of two genes encoding proteins with ankyrin domains, *ank2* and *pk1*, and one gene putative secreted protein gene, *GP15*, following the method of [40, 81]. Polymorphism of *ank2* and *pk1* markers was analyzed using RFLP analyses as described by [81], while polymorphism of *GP15* was examined through direct sequencing of PCR products. Examination of the allelic profile of these 3 markers allows the assignment of each individual infection to one of the five known *Wolbachia* groups in *Cx. pipiens* (named *w* Pip-I to *w* Pip-V [40]).

Filarial nematodes were detected using a PCR assay amplifying a fragment of the cytochrome c oxidase 1 gene (*CO1*) using the generalist primers COlintF and COlintR as described in [82]. DNA from a rodent filarial nematodes, *Litomosoides sigmodontis*, was used as positive control in each PCR assay. DNA sequencing of the obtained products was performed in reverse sense using COlintR and sequences have been deposited in GenBank.

### Insecticide resistance status

The insecticide resistant or susceptible status of the mosquitoes was carried out on the same random sample of individuals as above (n = 100 from the “haemosporidian pool” and n = 92 from the “control pool”). This was analyzed using RFLP analysis as described in [50]. This technique allows us to distinguish between 4 insecticide resistance status in *Cx. pipiens*: S (fully susceptible), E (resistant through the overproduction of the carboxylesterase, *Ester* gene), A (resistant through the modification of the acetylcholinesterase gene, encoded by *ace-1*) or AE (resistant through both acetylcholinesterase modification and esterase overproduction).

### Statistical analyses

Analyses were carried out using the R statistical package (v. 3.0.2). The prevalence of infected mosquitoes was analyzed on the whole dataset (n = 1156 mosquitoes) using GLM models with a binomial error structure, fitting mosquito sampling location (*site*), sampling time (*month*) and their interaction as fixed explanatory variables. The significant effect of *month* on mosquitoes infection prevalence was further confirmed by a mixed effect model approach in order to account for i) the nested structure of the dataset (*month* within *site*); and ii) potential temporal auto-correlations [83]. Parasite prevalence (calculated for each *site* x *month* combination) was arcsine square root transformed [83] prior to fitting in a lme model (nlme package) using *month* as a fixed effect, *site* as a random term, and adding a temporal autocorrelation structure (*month* within *site*, corAR1 function) to our model as described in [84]. All other analyses were performed on the subsample of 192 mosquitoes belonging either to the “haemosporidian” or “control” pools. Haemosporidian lineage richness was calculated as the total number of lineages encountered, while lineage diversity was calculated using the Shannon-Weaver index (*vegan* package). Richness and diversity were calculated for each *site* x *month* combination. GLM models with a normal error structure were used to test the effect of the following four explanatory variables on both richness and diversity: *site*, *month*, *sample size* (number of mosquitoes captured), and *infection prevalence* (proportion of Haemosporidian-infected mosquitoes). *Sample size* and *infection prevalence* were also estimated for each *site* x *month* combination. As there were not enough individuals of each insecticide resistant category to warrant separate analyses for each of them, all insecticide resistant mosquitoes were grouped within a single insecticide resistant class (giving a binomial *IR* response variable with two levels: resistant and susceptible). Similarly, only two *Wolbachia* subgroups were found in mosquitoes giving a binomial response variable (*Wolb*). The effect of *site* and *month* on the probability of being insecticide resistant (*IR*) or belonging to a given *Wolbachia* subgroup (*Wolb*) were therefore analyzed using GLM models with a binomial error structure. The effect of haemosporidian infection on *IR* or *Wolb* was analyzed using a mixed model procedure (lmer, lme4 package) with a binomial error structure, fitting *infection* as a fixed explanatory variable and *site* as a random explanatory variable.

The general procedure for building all statistical models was as follows. Maximal models were built including all high order interactions and were simplified by sequentially eliminating non-significant terms and interactions to establish a minimal model [83]. The significance of the explanatory variables was established using F-tests or *χ*^2^ tests for models with normal error structure or binomial error structure respectively. P = 0.05 was used as a cut-off p-value. The significant F or *χ*^2^ values given in the text are for the minimal model, whereas non-significant values correspond to those obtained before the deletion of the variable from the model. In models using a binomial error structure we systematically checked for over dispersion by calculating that the ratio of *residual deviance* over *residual degrees of freedom* was <2 [83]. All our models satisfied this premise. The full data-set is given in the Additional file 1.

## Results

### Infection prevalence

Of the 1156 *Cx. pipiens* mosquitoes that were captured at the four sampling sites (Figure 1), 9.52 ± 0.86% were found to be infected with haemosporidian parasites by nested PCR (henceforth ‘naive’ prevalence, see Methods and Table 2 for details). When we accounted for imperfect PCR detection (occurrence of false negatives) using site occupancy models, the estimated infection prevalence was found to be somewhat higher: 13.82 ± 0.82%. Only a single infected mosquito was collected in November so this time period was not included in subsequent analyses. The proportion of infected mosquitoes fluctuated across months, showing a distinctive “humped” pattern from April to August. Over the 9-month sampling period, however, the overall trend was that of a significant increase in prevalence: from 0% in February to 15.8% in October, (main *month* effect, χ^2^_1_ = 13.23, p < 0.001, see Figure 2). This effect remained significant when accounting for potential temporal autocorrelation patterns or when nesting sampling time within sampling sites (main *month* effect, χ^2^_1_ = 9.74, p = 0.002, autocorrelation term, χ^2^_1_ = 1.62, p = 0.20). These fluctuations in parasite prevalence were similar across the four sampling sites (main *site* effect, χ^2^_3_ = 0.63, p = 0.89; site * month, χ^2^_3_ = 4.84, p = 0.18).

**Table 2:** **Haemosporidian lineage occurrence and co-occurrence with** ***Wolbachia*** **and insecticide resistance in mosquitoes collected in Southern France in 2006**

	Haemosporidians lineages	Total number (proportion)	Sampling time	Sampling location				***w*** Pip groups		Insecticide resistance			
				SUS	TC	MEJ	MDV	II	III	S	E	A	AE
***Plasmodium***	SGS1	44* (3.81)	April to October	8	22	5	9	27	17	6	32		6
	DELURB4	21 (1.82)	April to October	1	9	7	4	9	12	5	14	1	1
	DELURB5	10* (0.87)	May to August, October		4	3	3	2	8	1	7		1
	PADOM01	7 (0.61)	July, August	1	2	4		2	5	1	4		2
	GRW06	3 (0.26)	August, September		2		1	1	2		2		1
	SYAT05	3 (0.26)	April, June		2	1			3		3		
	COLL1	1 (0.09)	May			1		1			1		
	CXPIPS1	1 (0.09)	August		1			1			1		
	CXPIPS2	1 (0.09)	August	1				1					1
	GRW11	1 (0.09)	September		1			1			1		
	LINN1	1 (0.09)	September		1				1	1			
	PADOM01 like	1 (0.09)	June		1			1			1		
	SGS1 like	1 (0.09)	June		1				1		1		
***Haemoproteus***	CXPIPS3	2 (0.17)	May			1	1	1	1		1		
	GAGLA03	1 (0.09)	August	1				1			1		
**Total number of mosquitoes tested**		96 sequenced		149	543	299	165	89	102	26	138	1	23

A total of 1156 mosquitoes were sampled for this study. Lineages are given for each haemosporidian genus: *Plasmodium* and *Haemoproteus*. Asterisks represent multiple infections (two mosquitoes were infected by both SGS1 and DELURB5). The number of infected mosquitoes is given for each sampling location (SUS, Sussargues; TC, Tour Carbonnière; MEJ, Méjanes; MDV, Marais du Vigueirat), *Wolbachia* group, and insecticide resistance status (S, fully susceptible; E, resistant through carboxylesterase overproduction; A, resistant through acetylcholinesterase modification; AE, resistant through both acetylcholinesterase modification and esterase overproduction).

### Richness and diversity of haemosporidian lineages

Of the 96 haemosporidian–infected mosquitoes whose *cytb* gene was sequenced, 92 contained single infections. In the four remaining cases, chromatograms showed double nucleotide peaks indicating mixed infections by different hemosporidian lineages. In two of these cases, it was possible to identify the peaks as being a mixture of SGS1 and DELURB5. In the other two cases, however, it was not possible to identify the combination of lineages and they were therefore taken out from the analyses.

**Figure 3:**
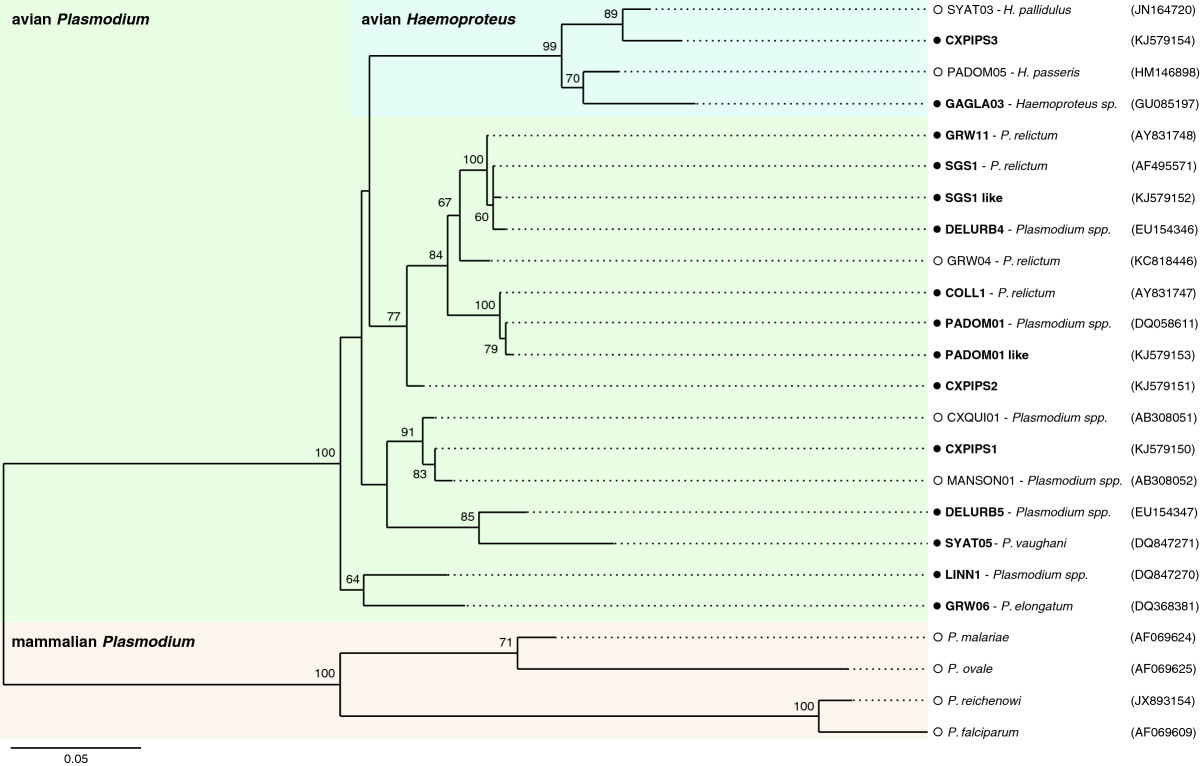
**Phylogenetic relationships of haemosporidian parasites isolated from mosquitoes (**●**) and other reference malaria parasites (○** **) based on the cytochrome b (448 bp).** Haemosporidian sequences from *Cx. pipiens* mosquitoes sampled in this study are shown in bold. Branches having bootstrap support (5000 replicates) with values under 60% are omitted. Scale bar indicates number of nucleotide substitutions per site. “like” means that the strain isolated in this study differs only by 1 bp from the reference sequence. Morphospecies or parasite genuses are given for lineages referenced in GeneBank and using the MalAvi database.

Overall, the analysis led to the identification of 15 different haemosporidian lineages, 13 of which belonged to the *Plasmodium* genus and two to the *Haemoproteus* genus (see Table 2, Figure 3). Thirteen of the haemosporidian lineages found in this study clustered with previously known lineages (see Table 1). Three other lineages were found for the first time and could not be assigned to a given morphospecies, though two of them unambiguously fell within the *Plasmodium* genus and the third one within the *Haemoproteus* genus. The first one, which we named CXPIPS2 is close to several lineages within the *P. relictum* morphospecies (e.g. 97% identity at the nucleotide level with SGS1, GRW11 and COLL1; cf. Table 1 and Figure 3 for GenBank accession nos), while the second one, CXPIPS1, is closest to CXQUI01 (99% identity) and MANSON01 (99% identity) lineages which have been isolated in Japanese mosquitoes (*Cx. quinquefasciatus* and *Mansonia sp.* respectively [72]). Finally, the CXPIPS3 sequence matches that of several *Haemoproteus* lineages such as *H. pallidulus* lineage SYAT03 (97% identity), and *H. passeris* lineage PADOM05 (95% identity).

**Figure 4:**
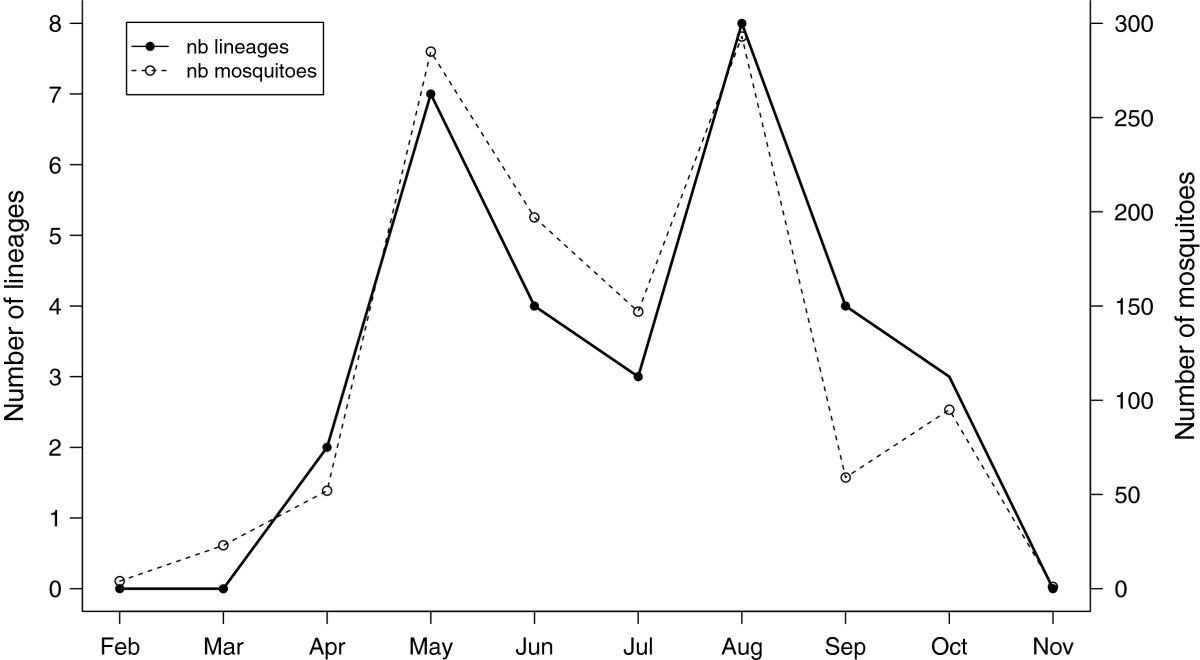
**Number of mosquitoes and haemosporidian lineage richness over time.** The number of mosquitoes over the ten-month sampling period is plotted as empty circles; the number of haemosporidian lineages (lineage richness) is plotted as black-filled circles over the same sampling period.

There was a considerable fluctuation in the number of lineages (lineage richness) present in mosquitoes across the 10-month study (see Figure 4, Table 2) with the maximum richness (7–8 lineages) happening in late spring (May) and summer (August). This fluctuation across time, which was independent of the sampling site, was only marginally non-significant (*month* effect: F_1,25_ = 3.94, p = 0.058, *site* effect: F_3,23_ = 0.32, p = 0.81). The diversity of these lineages (estimated by the Shannon-Weaver index), on the other hand, was not dependent on either the time (F_1,20_ = 0.75, p = 0.40) or the location of sampling (F_3,18_ = 0.68, p = 0.57). Both haemosporidian richness and diversity were best predicted by the interaction between the number of mosquitoes captured and the prevalence of infected mosquitoes (significant *prevalence x sample size* interaction for lineage richness: F_1,24_ = 8.12, p = 0.009; for lineage diversity: F_1,19_ = 4.89, p = 0.040).

### Effect of Insecticide resistance, *Wolbachia* and filarial nematodes on *Plasmodium* prevalence

**Figure 5:**
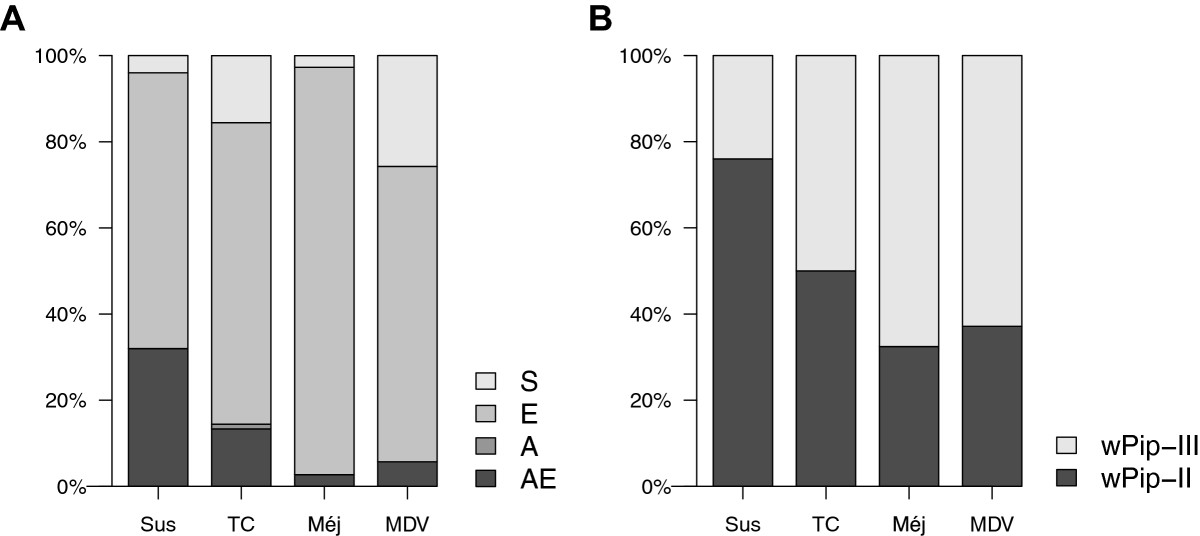
**Sampling site effect on insecticide resistance and** ***Wolbachia*** **-infection. (A)** Barplot of the proportion of insecticide susceptible (S) or resistant *Culex pipiens* mosquitoes (E, A or AE) in each site; **(B)** Barplot of the proportion of *Culex pipiens* mosquitoes infected with either *w* Pip II or *w* Pip III *Wolbachia* in each of the 4 sampling sites (Sus: Sussargues, TC: Tour Charbonnière, Méj: Méjanes, MDV: Marais du Vigueirat).

**Figure 6:**
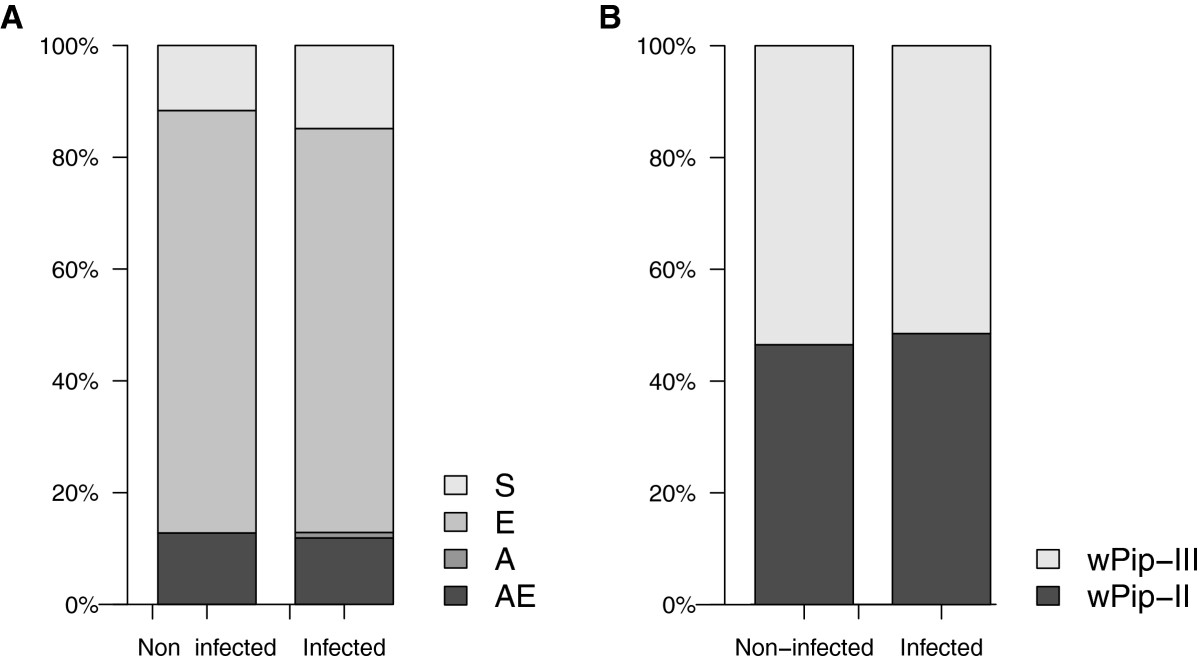
**Haemosporidian infection,** ***Wolbachia*** **infection and insecticide resistance. (A)** Barplot of the proportion of insecticide susceptible (S) or resistant *Culex pipiens* mosquitoes (E, A or AE) in Haemosporidia*-* infected and -uninfected mosquitoes; **(B)** Barplot of the proportion of *Culex pipiens* mosquitoes infected with either *w* Pip II or *w* Pip III *Wolbachia* in Haemosporidia*-* infected and -uninfected mosquitoes.

Of the 192 mosquitoes used for this analysis, 86.6% were found to be insecticide resistant through known mutations either at the *ace-1* locus (A: 0.5%), the *Ester* locus (E: 73.8%), or both loci at the same time (AE: 12.3%; Table 2). The site at which mosquitoes were captured was a significant predictor of insecticide resistance status (χ^2^_3_ = 11.83, p = 0.008, see Figure 5A) while sampling time had no effect (χ^2^_1_ = 0.02, p = 0.89). Haemosporidian infection in mosquitoes was not correlated with their insecticide resistance status (χ^2^_1_ = 0.63, p = 0.43, see Figure 6A).

*Wolbachia* infection was detected in the 192 examined specimens, as expected from results of previous studies showing that infection is fixed in *Cx. pipiens* populations [36, 37, 85]. The analysis of allelic profiles of three diagnostic *Wolbachia* genes revealed that 47% of these mosquitoes were infected with bacteria from the *w* Pip-II group while the remaining 53% were infected with the *w* Pip-III group (Table 2). As observed for insecticide resistance, the only significant predictor of *w* Pip group was the site at which mosquitoes were captured (χ^2^_3_ = 13.91, p = 0.003, see Figure 5B) while sampling time had no effect (χ^2^_1_ = 0.23, p = 0.63). Haemosporidian infection in mosquitoes was also not correlated with the *w* Pip group they were infected with (χ^2^_1_ = 0.12, p = 0.73, see Figure 6B).

Infection by filarial nematodes was rare: it concerned only two of 192 individuals (1%), both of which were in the *Plasmodium*-infected group (coinfections with the DELURB5 and CXPIPS2 lineages). The two *CO1* filiarial sequences were strictly identical and clearly match with sequences from members of the Onchocercidae family. The *CO1* sequences obtained in this study [GenBank: KJ612514] were however not identical to sequences available in GenBank, preventing us from identifying the species infecting our *Cx. pipiens* samples. The most closely related sequence (90.4% identity at the nucleotide level) found in GenBank is from the avian filarial nematode *Chandlerella quiscali* [GenBank: HM773029], which is naturally found in several species of Passeriformes.

## Discussion

### Infection prevalence and diversity

The high prevalence of *Plasmodium* spp. found in wild-caught *Cx. pipiens* in this study confirms the important role of this mosquito species as the main vector of avian malaria in the European continent [4, 6, 7, 10], although definite proof of its role requires further experimental efforts. The prevalence of *Plasmodium spp* found in *Cx. pipiens* (9.5% naive and 13.8% estimated), is roughly of the same magnitude as that observed in a Swiss forest in the same period (6.6-16.6%; [4, 6]). Remarkably, this also falls within the range observed in wild-caught *Anopheles* mosquitoes infected with human *Plasmodium* parasites (ca. 10-15% of infected mosquitoes, e.g. [86, 87]), suggesting that different malaria parasites may be ultimately driven by similar biological constraints [88].

We observed a marked seasonal variation in avian malaria parasite prevalence in mosquitoes, as has also been documented in birds [73, 89], and, recently, in mosquitoes from Switzerland [4] and Spain [11]. Avian parasites were not observed in February-March, but from April to October the prevalence increased from ca. 3.9% to ca. 15.8%. This is consistent with the biology of European populations of *Cx. pipiens* which are known to go into diapause in winter and to cease blood feeding from October to March [90]. The mosquitoes collected at the beginning of the season (February, March) were thus either old overwintered mosquitoes, or their first descendants. Either way, the lack of avian *Plasmodium* infections in mosquitoes at the beginning of the season proves that the parasite’s winter reservoir is the bird and not the mosquito host [73, 91]. *Plasmodium relictum* is indeed unlikely to survive the overwintering period within mosquitoes as prolonged exposures to low (ca. 12-15°C) temperatures are shown to be lethal for the parasite developing within mosquitoes [92, 93]. In contrast, infections in the bird are characterized by an initial (acute) phase with high parasitaemias, followed by a low level (chronic) phase which can last for months or even years [94–96]*.* It therefore follows that the rise in malaria prevalence observed from April onwards must be the result of *de novo* infections following the first infected blood meals at the beginning of the season. From here on, prevalence increases almost linearly until October. Worthy of note is that although *Cx. pipiens* constituted over 90% of the mosquitoes collected in our sampling, other mosquito species were also present [54], some of which may play a role in the transmission of avian haemosporidians [12] and thus in the overall dynamics of infections.

Our results reveal that avian malaria parasites are very diverse in *Cx. pipiens* females, as also recently pointed out by recent studies [1, 4, 6]. Fifteen different lineages were obtained amongst the 96 mosquitoes that tested *Plasmodium*-positive, all of which have been described as being either partially or exclusively parasites of Passeriform birds (Table 1). The most prevalent lineage was SGS1 (44.90% of the lineages found), confirming its status as the most abundant haemosporidian lineage in Europe [4, 6, 95], followed by DELURB4 (21.43%) and DELURB5 (10.20%). All lineages identified here are known to infect both migratory and sedentary Passeriforms (Table 1), except DELURB5, which has, to date, only been sampled from a migratory bird, the common house martin (*Delichon urbicum*), in Spain [66]. Interestingly, the highest richness in terms of the number of lineages happens in the summer, coinciding with both the period of high vector abundance, a common pattern in haemosporidia [97], and with the breeding period of migratory birds in the study area.

The high diversity of lineages in *Culex pipiens* mosquitoes suggests that such generalist vectors [62, 98, 99] may play an important role in the high frequency of host switching that characterizes avian malaria, and which can sometimes take place across great host taxonomic distances [100]. Yet the factors allowing the maintenance of the coexistence between multiple malaria lineages must be considered. For instance, migratory birds may also play a role in the maintenance of this diversity [63, 101, 102]. For instance, Waldenstrom *et al.*[63] have evidenced that resident African birds can exchange African haemosporidian local lineages with European migrant birds, highlighting the role of bird migration in *Plasmodium* distribution. A recurrent problem of parasite prevalence estimations from wild caught mosquito samples is the potential for either overestimating or underestimating the proportion of infectious mosquitoes in the population. Overestimation comes about from the assumption that all PCR-positive samples translate into a vector-competent mosquito. Parasites present in the blood meal may however fail to establish a viable infection in mosquitoes and may be subsequently eliminated. Leftovers from previous infected blood meals containing parasite DNA residues may lead to PCR amplifications [13]. In addition, even when parasites are seen to be infecting certain vector tissues (e.g. oocysts in the midgut), this does not necessarily imply that the parasite will complete its intrinsic incubation cycle all the way to the transmissible (sporozoite) stages in the salivary glands [103]. Examples of such ectopic parasite development have been already reported in the literature [104, 105]. To avoid these pitfalls, we sampled our mosquitoes using bird-baited traps, under the assumption that mosquitoes searching for a blood meal have entirely digested previous blood meals, an assumption confirmed by the visual inspection of the mosquitoes’ abdomens prior to freezing. We cannot, however, exclude the possibility that some haemosporidian lineages detected in this study are not transmitted by *Cx. pipiens.* Such may be the case for the two *Haemoproteus* lineages (CXPIPS3 and GAGLA03) found in our samples. Other studies have also detected *Haemoproteus* parasites in field-caught mosquitoes [9, 10, 13]. *Haemoproteus* parasites are, however, currently thought to be transmitted exclusively by biting midges [106]. Experimental evidence available, albeit limited, indeed seems to exclude mosquitoes as vectors [105, 107].

Underestimation, on the other hand, constitutes a more pervasive problem as it comes about through difficulties in diagnosing haemosporidian infection using the nested-PCR method. Indeed, because detection rates are strongly dependent on the relative concentrations of parasites and total DNA within the extract [17, 18], low parasite loads go through undetected (i.e. false negatives). Although seldom used in the context of host-parasite interactions, site-occupancy modelling is an efficient means to examine the accuracy of naive estimates of organismal prevalence and for determining potential sources of detection bias [17]. Gomez-Diaz *et al*. [17] advocate the inclusion of such models for all pathogen survey techniques. Here, the application of site occupancy models leads us to increase our initial (“naive”) estimate of *Plasmodium* prevalence in mosquitoes by over 4%. An accurate estimation of the proportion of infectious mosquitoes in a population is essential to estimate the level of exposure of hosts to the parasite within a population (the “entomological inoculation rate”), a key epidemiological tool to estimate malaria endemicity and transmission intensity within a host population [108].

### Effect of *Wolbachia* co-infections on *Plasmodium* prevalence, richness and diversity

Although an enormous amount of effort has gone into investigating the interaction between *Wolbachia* endosymbionts and a range of parasites, our study is, to our knowledge, the first investigation of *Wolbachia-Plasmodium* interactions in field-caught mosquitoes. *Wolbachia* infections are fixed in wild *Cx. pipiens* mosquito populations worldwide [36, 37], so it is not possible to compare the prevalence of *Plasmodium* in *Wolbachia*-infected and uninfected mosquitoes. However, *Wolbachia* infections are polymorphic within *Cx. pipiens* populations, with individual mosquitoes being infected by one of the five known *Wolbachia w* Pip groups [40, 81]. In our sampling area, we identified two of these groups: *w* Pip-II and *w* Pip-III, both of which are commonly found in the Northern hemisphere [40, 81]. The frequency of these two *w* Pip groups varied between the four study sites, with *w* Pip-II being more frequent in the western populations (76% in Sussargues) and less frequent in eastern populations (roughly 34% in Méjanes or in Marais du Vigueirat, Figure 5B). This result agrees with previous work done in this region, showing geographic differences in the frequency of the different *Wolbachia* groups [37, 39]. While in other mosquito species previous work has shown that the effect of *Wolbachia* on *Plasmodium* development is strain-specific [29–35], here, neither *Plasmodium* prevalence nor diversity was affected by the *w* Pip group co-infecting *Cx. pipiens* females. This result thus suggests that these two groups of *Wolbachia* have either no effect on *Plasmodium* development [109] or act in the same way (i.e. inhibit [29–33] or facilitate [32, 34, 35] the parasite’s development). Although a *Wolbachia* strain from the wPip-III group has been shown to facilitate *P. relictum* SGS1 infection in *Cx. pipiens*[35], further experimental work, using strains from both *Wolbachia* groups and different *Plasmodium* lineages would be necessary in order to generalize this pattern.

### Effect of filarial co-infections on *Plasmodium* prevalence and diversity

While *Culex* mosquitoes are vectors of a wide range of filarial parasites [42–44, 110], little is known about the outcome of concomitant filarial-*Plasmodium* infections within the vector. Remarkably, previous studies on non-*Culex* species have shown that simultaneous transmission of the two parasites is particularly rare in the field, suggesting that competition is likely to be a common outcome in nature [111, 112]. Here, we only found two mosquitoes (1%) infected with an unknown avian filaria parasite, even though birds are known to be often infected with multiple filarial species [113–115]. The observed avian filarial parasite is closely related to *Chanderella quiscalli*, a species known to parasitize the brain of various Passeriform birds. Both of these filaria-positive mosquitoes were found in the *Plasmodium*-positive pool, one in co-infection with DELURB5 (and with the *w* Pip3 *Wolbachia* group) and the other one with CSPIPS2 (and with *w* Pip2). These results agree with data obtained from *Cx. pipiens* mosquitoes in Germany, where an unidentified avian filarial parasite, very close to *C. quiscalli*, was also found at very low prevalence [110]. Overall, our data suggest that filarial parasites may be not common enough to have a significant effect on the population dynamics of avian *Plasmodium* parasites in this region.

### Effect of insecticide resistance on *Plasmodium* prevalence and diversity

Despite extensive knowledge on the intimate physiological relationships existing between *Plasmodium* and mosquitoes on the one hand, and on the physiological consequences of insecticide resistance for the mosquito on the other, these two questions have, surprisingly, rarely been put together to ask whether the evolution of insecticide resistance can affect the transmission of *Plasmodium* (but see [50, 51, 116])*.* Insecticide resistance could interfere with *Plasmodium* development in at least two ways [47]. First, the physiological modifications that accompany the deployment of insecticide resistance mechanisms may render the vector toxic to parasites. Second, insecticide resistance could affect vector immunity. In one of the few studies to have explicitly investigated the connection between insecticide resistance and disease transmission, McCarroll and collaborators showed that the development of the filaria *Wuchereria bancrofti* larvae was arrested in insecticide-resistant *Cx. quinquefasciatus* mosquitoes [48, 49]. Exactly what rendered the insecticide-resistant mosquito toxic to the parasite is not known, but it was hypothesised that the overproduction of carboxylesterases in these mosquitoes resulted in a change in the redox potential of the tissues hosting the parasite, which led to the death of the larvae. Experimental infections carried out in the laboratory have rendered contradictory results [50, 116]. To our knowledge, no study exists that investigates the impact of insecticide resistance on *Plasmodium* prevalence in naturally infected wild-caught mosquitoes. Following repeated treatments of larval sites with organophosphate insecticides (initiated 40 years ago), it is therefore not surprising that insecticide resistance was found in the *Cx. pipiens* populations examined. However, that the overwhelming majority of the mosquitoes sampled (86.6%) were found to be insecticide resistant was somewhat of a surprise, given that in this region classic (organophosphate) insecticides were substituted in 2006 by Bti (*Bacillus thuringiensis israelensis*[117]). The consequences of these high resistance levels for the circulation of pathogens such as West Nile virus, which has been known to cause episodic cases in both humans and animals in the region, need further study [118]. The high prevalence of insecticide resistance through carboxylesterase overproduction (a general detoxification mechanism) may be the result of pollutant transfer from neighbouring agrosystems. We found no effect of insecticide resistance on avian malaria prevalence or diversity. However, the low frequency of insecticide-susceptible mosquitoes in the sample considerably reduced the statistical power to detect differences between resistant and susceptible mosquitoes. Further work should consider sampling *Cx. pipiens* populations along several North–south transects spanning populations with low (North) and high (South) recorded frequencies of insecticide resistant genes [119].

## Conclusions

In conclusion, we found that haemosporidian parasites are common and diverse in wild-caught *Cx. pipiens* mosquitoes in Southern France, but that their prevalence is independent of the co-infection and insecticide resistant status of the vector. A correct estimation of the prevalence of infected mosquitoes in a population is essential in order to understand the epidemiology of the disease. The application of site occupancy models leads to a considerable increase in our estimates of *Plasmodium* prevalence in mosquitoes, suggesting that imperfect detection should be taken into account in further studies. Our study fails to detect spatial variations in prevalence among sampling sites. Yet, we confirm the existence of a temporal pattern where malaria prevalence increases throughout the season. This temporal trend strongly suggests that the bird population is used as a reservoir of avian malaria during the winter. The fact that multiple sedentary or migratory bird species may host *Plasmodium* parasites during the winter may explain the maintenance of the diversity of malaria lineages in the Southern France.

## Electronic supplementary material

Additional file 1: **Complete dataset.** Haemosporidian naïve prevalence (0 or 1), corrected prevalence (based on repeated PCRs results, 0,0549 to 1), lineage identity and the occurrence of co-infections are given for *Culex pipiens* mosquitoes collected between February and December 2006 in 4 different sampling sites (Sus: Sussargues, TC: Tour Charbonnière, Méj: Méjanes, MDV: Marais du Vigueirat). The “pool” columns indicate the data subset used for (a) phylogenetic analyses, (b) diversity index and species richness calculation, and (c) the insecticide resistance and *Wolbachia* subgroup prevalence according to different covariates (*site, month* and *haemosporidian prevalence*, see Methods). *Wolbachia* groups (wolb, wPip2 or wPip3) and insecticide resistance status (S: fully susceptible, E: overproduction of carboxylesterase, A: acetylcholinesterase modification, AE: both acetylcholinesterase modification and esterase overproduction) are indicated for all mosquitoes that were included in these analyses. Other abbreviations used in the table are ‘ - ’ (not studied), ‘ 0 ’ (absence), ‘ 1 ’ (presence), and ‘ na ’ (failed). (XLSX 224 KB)
